# Local viscoelasticity of living cells measured by rotational magnetic spectroscopy

**DOI:** 10.1038/ncomms10134

**Published:** 2016-01-05

**Authors:** J.-F. Berret

**Affiliations:** 1 Matière et Systèmes Complexes, UMR 7057 CNRS Université Denis Diderot Paris-VII, Bâtiment Condorcet, 10 rue Alice Domon et Léonie Duquet, 75205 Paris, France

## Abstract

When submitted to a magnetic field, micron-size wires with superparamagnetic properties behave as embedded rheometers and represent interesting sensors for microrheology. Here we use rotational magnetic spectroscopy to measure the shear viscosity of the cytoplasm of living cells. We address the question of whether the cytoplasm is a viscoelastic liquid or an elastic gel. The main result of the study is the observation of a rotational instability between a synchronous and an asynchronous regime of rotation, found for murine fibroblasts and human cancer cells. For wires of susceptibility 3.6, the transition occurs in the range 0.01–1 rad s^−1^. The determination of the shear viscosity (10–100 Pa s) and elastic modulus (5–20 Pa) confirms the viscoelastic character of the cytoplasm. In contrast to earlier studies, it is concluded that the interior of living cells can be described as a viscoelastic liquid, and not as an elastic gel.

## Abstract (web summary)

Cells are recognized as having viscoelastic properties, but whether the cytoplasm resembles a viscoelastic liquid or an elastic gel is still debated. Here the authors use micron-sized wires rotating at variable speeds to show that the cytoplasm has properties of a viscoelastic liquid.

## Graphical abstract

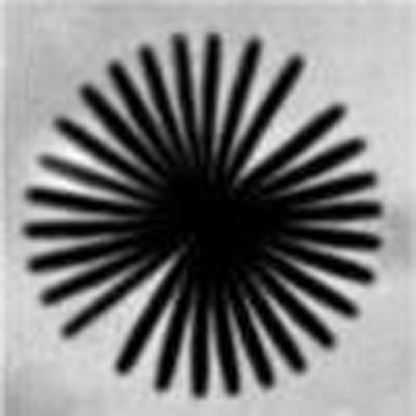

In rheology, viscoelastic liquids and viscoelastic solids differ from each other by their stress relaxation function *G*(*t*). In viscoelastic liquids, *G*(*t*) is a decreasing function of the time which tends to zero as *t*→∞, whereas in viscoelastic solids *G*(*t*) tends to a finite elastic modulus, indicating the presence of residual unrelaxed stresses even at very long time durations^1^. Hence, the static shear viscosity, which is defined as 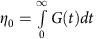 takes a finite value for liquids and it is generally expressed as a product of a relaxation time and of an elastic modulus. For viscoelastic solids, the static viscosity is not defined, and the rheological properties are described in terms of a low frequency elastic modulus *G*′(*ω*→0) and of a yield stress, that is, the critical value of the stress above which the sample does flow. In the following, viscoelastic solids will be termed ‘elastic gels' for convenience. Here we address the question of the rheological properties of the intracellular medium of mammalian cells, and in particular the question to know whether the cytoplasm of such cells is a viscoelastic liquid or an elastic gel.

Rheometers are used to determine the relationship between the strain and stress on material sample of volume of the order of millilitres. Microrheology in contrast uses micron-size probes embedded in the material and needs much less sample, of the order of 1 picolitre. The past 20 years have seen increasingly rapid advances in this field, specifically in cell and tissue biomechanics^2^,^3^. For living cells, techniques including atomic force microscopy (AFM)^4^,^5^, optical and magnetic tweezers^6^,^7^,^8^,^9^,^10^, parallel microplates^11^ and active tracking of internalized particles^7^,^10^,^12^,^13^,^14^ were developed and provide consistent evaluation of the time or frequency dependencies of rheological parameters, such as the elastic compliance *J*(*t*) or of the complex modulus G*(*ω*). Numerous studies performed on a wide variety of cell lines have shown that *J*(*t*)^9^,^14^,^15^ and G*(*ω*)^8^,^9^,^12^,^14^,^16^ obey scaling laws as a function of time or frequency, with exponents comprised between 0.1 and 0.5. As for G*(*ω*) measurements, the elastic modulus *G*′(*ω*) was found to be larger than the loss modulus *G*′′(*ω*) on broad frequency ranges. These results were interpreted as an indication that the mechanical response of the cell or of the cytoplasm is that of a weak elastic solid^8^,^9^,^10^,^12^,^15^,^17^, or in the terminology adopted previously that of an elastic gel. Out-of-equilibrium modelling suggests that the cell interior is a kind of soft glassy material with an effective temperature higher than that of the physiological temperature^17^. Alternative approaches of biomechanics were also attempted and provided different view points. On the basis of AFM micro-indentation and force-relaxation tests, Moeendarbary *et al.*^5^ proposed that the cytoplasm behaves as a poroelastic material, where the water redistribution within the cytoplasm plays a fundamental role in setting the internal rheology. Other studies, in particular the earlier work of the Sackmann group^6^,^7^,^18^ suggest that the intracellular medium behaves like a liquid and is characterized by a finite value of the shear viscosity^13^. More recently, Kuimova *et al.*^19^,^20^ developed porphyrin-dimer-based molecular rotors in which fluorescence emitting frequencies scale with the viscosity of the surrounding medium. Evaluated on immortalized cervical cancer cells (HeLa cells), these authors found intracellular viscosities in the range 0.05–0.2 Pa s. Chevry *et al.* show that micron-size wires internalized in mouse fibroblasts and MDCK epithelial cells exhibit Brownian-like orientational fluctuations, associated to finite effective viscosity of 0.1–1 Pa s, in relative agreement with the determinations of Kuimova *et al.*^21^.

Regarding the complexity of the dynamics in living systems, and the contrasting results obtained so far, there is a need to develop new types of probes and protocols for testing the cell biomechanics. Following the pioneer work by Crick and Hughes some 60 years ago^22^, recent studies have shown that microrheology based on the tracking and monitoring of anisotropic probes could bring significant advances to the field^23^,^24^,^25^,^26^,^27^,^28^,^29^,^30^,^31^,^32^,^33^,^34^. It has been proposed, for example, that the shear viscosity of a fluid could be determined from the motion of a micro-actuator submitted to a rotating electric^28^ or magnetic field^27^,^32^,^33^,^34^,^35^,^36^. These techniques are described as electric or magnetic rotational spectrometry^29^,^30^. With micro-actuators rotating at increasing frequency, a transition between a steady and a hindered motion is predicted for viscous fluids, and it is found experimentally^25^,^27^,^29^,^30^,^37^,^38^. A recent quantitative analysis shows that for viscoelastic liquids, the elasticity does not affect the onset of the instability and that the expression for the critical frequency remains the same as in a viscous fluid^25^. For an elastic gel of infinite static viscosity, in contrast, extrapolation yields a critical frequency going to zero, suggesting that the technique of the rotating wire is well adapted to differentiate liquid- from gel-like behaviours, as defined in rheology.

In the present paper, we exploit this concept to study the mechanical response of the cytoplasm of mouse fibroblast NIH/3T3 and human cancer HeLa cells. It is first demonstrated that the micron-size wires specifically fabricated for the study are nontoxic, enter spontaneously inside living cells and that they are not comprised in membrane-bound compartments. Actuated by a low-frequency external field, the wires rotate at the same angular speed than the field, whereas at higher frequency their motion exhibits back-and-forth oscillations. Values for the static viscosity, elastic modulus and relaxation time of the cytoplasm are derived from a complete analysis of the wire temporal trajectories. It is concluded that the interior of fibroblasts and HeLa cells can be appropriately described as viscoelastic liquids.

## Results

### Magnetic wire synthesis and characterization

**Figure 1:**
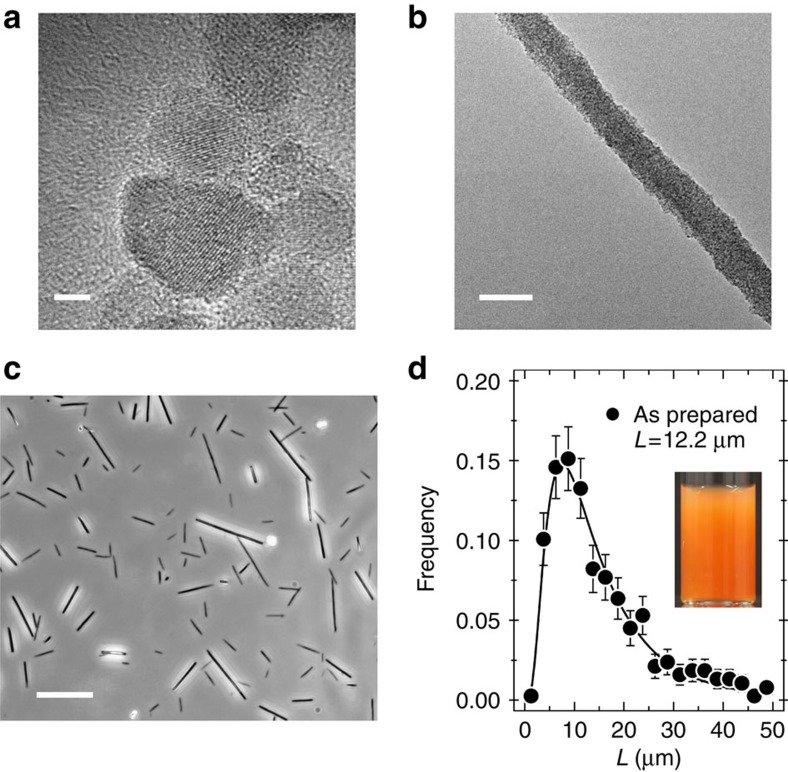
Characterization of particles and wires. (**a**) High-resolution transmission electron microscopy (TEM) of 13.2 nm iron oxide nanoparticles (scale bar, 2 nm). (**b**) TEM of magnetic wires made by co-assembly using oppositely charged particles and polyelectrolytes (scale bar, 200 nm). (**c**) Phase-contrast image of magnetic wires deposited on a glass substrate and observed by optical microscopy (× 60, scale bar, 10 μm). (**d**) Size distribution of the wires adjusted using a log-normal distribution function with length of 12.2 μm and dispersity of 0.70. Error bars are defined as s.e.m. Inset: image of a vial containing a dispersion of wires at the concentration of 10^6^ wires μl^−1^.

Crystalline iron oxide nanoparticles were synthesized by co-precipitation of iron(II) and iron(III) salts in alkaline aqueous media and by further oxidation of the magnetite (Fe_3_O_4_) into maghemite (γ-Fe_2_O_3_). The size and dispersity (ratio between standard deviation and average diameter) of the particles prepared were determined from transmission electron microscopy (*D*_TEM_=13.2 nm, *s*=0.23, Fig. 1 and Supplementary Fig. 1). Their crystallinity and structure was assessed by electron beam microdiffraction (Supplementary Fig. 2). The magnetization curves at different volume fractions were obtained by vibrating sample magnetometry and adjusted using a paramagnetic model^39^,^40^. The adjustment provided a specific magnetization of 3.5 × 10^5^ A m^−1^ and a magnetic diameter of 10.7 nm (Supplementary Fig. 3). Light scattering was used to measure the weight-average molecular weight (*M*_*w*_=12 × 10^6^ g mol^−1^) and the hydrodynamic diameter (*D*_H_=27 nm) for the bare particles^40^. Values of the electrophoretic mobility and zeta-potential were also derived (*μ*_E_ =−3.8 × 10^−4^ cm^2^ V^−1^, *ζ*=−48 mV, Supplementary Table 1) and indicated that the dispersions were stabilized by electrostatic forces^41^.

For the wire synthesis, the particles were coated with poly(sodium acrylate)^39^,^41^ (Aldrich) of molecular weight *M*_*w*_=5,100 g mol^−1^. The wires were made according to a bottom-up co-assembly process using γ-Fe_2_O_3_-coated particles and oppositely charged polyelectrolytes. The polymer used was poly(trimethylammoniumethylacrylate)-b-poly(acrylamide) with molecular weights 11,000 g mol^−1^ for the cationic block and 30,000 g mol^−1^ for the neutral block^42^,^43^. Supplementary Fig. 4 illustrates the protocol for the fabrication of the wires. Figure 1b displays a TEM image of a single wire illustrating its nanostructure, whereas Fig. 1c shows a collection of wires deposited on a glass substrate and observed by optical microscopy (40 ×). The as-prepared samples contained anisotropic objects of median length 12.2 μm and of diameter 0.49 μm (Fig. 1d). Before incubation with cells, the wires were sonicated for 120 s to shorten their lengths and sorted by magnetic separation. The sonicated wires had an average length of 2.4 μm and a dispersity of 0.35. The wires dispersion was then autoclaved (Tuttnauer Steam Sterilizer 2340M) at 120 °C and atmospheric pressure for 20 minutes to prevent bacterial contamination for cell culture, concentrated by magnetic sedimentation at 10^6^ wires μl^−1^ and stored at 4 °C in a secure environment before use.

### Wire microrheology on model fluids and gels

First we derive the equation of motion of a wire immersed in well-known rheological models^1^ (Supplementary Fig. 5) and submitted to a rotating magnetic field, and then assess the validity of the technique by comparing the predictions to observations obtained on a wormlike surfactant solution and on a polysaccharide gel.

For a viscous liquid of viscosity *η*_0_, a wire submitted to a rotating field experiences restoring a viscous torque that slows down its rotation. As a result, its motion undergoes a transition between a synchronous and an asynchronous rotation. The critical frequency *ω*_C_ between these two regimes reads:


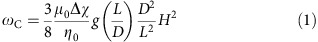


where *μ*_0_ is the permeability in vacuum, *L* and *D* the length and diameter of the wire, *H* the amplitude of the magnetic excitation and 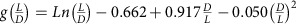 is a dimensionless function of the anisotropy ratio^44^,^45^. In equation (1), Δ*χ*=*χ*^2^/(2+*χ*) where *χ* denotes the magnetic susceptibility^27^. For data treatment, the geometrical characteristics are combined into the dimensionless parameter 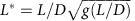. Under these conditions, equation (1) becomes:


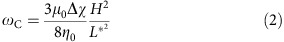


The average angular velocity Ω(*ω*) in the two regimes expresses as^27^,^35^:


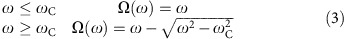


With increasing frequency, the average velocity increases linearly, passes through a cusp-like maximum at the critical frequency and then decreases. The transition between the synchronous and asynchronous regimes was used to calibrate the wire rheometer and determine the susceptibility parameter Δ*χ*. Experiments were performed at *T*=26 °C on a 85 wt. % water–glycerol mixture of static viscosity *η*_0_=0.062 Pa s^−1^. In a purely viscous fluid, the critical frequency is found to decrease as 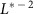 in agreement with equation (2). For wires made from 13.2 nm particles and block copolymers, we found Δ*χ*=2.3±0.7, and a magnetic susceptibility *χ*=3.6±0.9 (Supplementary Fig. 6). Here and in the following, the uncertainty is defined as s.e.m.

*A* Maxwell fluid is described by a spring and dashpot in series^1^. An actuated wire immersed in such a medium experiences a viscous and an elastic torque that both oppose the applied magnetic torque. The differential equation describing the wire motion has been derived and solved, leading to the following predictions (Supplementary Note 1). With increasing *ω*, the wire undergoes the same type of transition as the one described previously and the critical frequency *ω*_C_ expresses as in equation (1). The static viscosity *η*_0_ in equation (1) is however replaced by the product *Gτ*, where *G* and *τ* denote the shear elastic modulus and the relaxation time of the fluid. The set of equations in equation (3) is also identical to that of a Newtonian fluid. From the amplitude of the oscillations *θ*_B_(*ω*) (more precisely the angle by which the wire returns back after a period of increase) in the asynchronous regime, it is possible to determine the shear elastic modulus *G* using^25^:


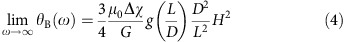


The above predictions were tested by monitoring the wire motion in a wormlike micellar solution made from cetylpyridinium chloride and sodium salicylate ([NaCl]=0.5 M) at 2 wt. % (ref. 46). The rheological parameters of the surfactant solution as determined by cone-and-plate rheometry were *η*_0_=1.0±0.1 Pa s, *G*=7.1±0.1 Pa and *τ*=0.14±0.01 s (Supplementary Note 2). From the evolution of the average angular velocity Ω(*ω*) of actuated wires and from the position of the maximum (equation (3)), a static viscosity of 1.3±0.3 Pa s was obtained. From the oscillation amplitudes in the regime 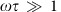 (equation (4)), a modulus of 9.4±2 Pa was derived (Supplementary Fig. 7). Experiments performed with wires of different lengths and in various magnetic field conditions confirmed the good agreement with cone-and-plate rheometry, and demonstrates the ability of the technique to measure the linear viscoelasticity of a Maxwell fluid^25^.

*A* Kelvin–Voigt element aims to describe a viscoelastic solid, and it is represented by a spring and dashpot in parallel^1^. The resolution of the differential equation of motion of a wire leads to the following predictions (Supplementary Note 1). At all frequencies, the wire rotation exhibits back-and-forth oscillations at a frequency twice that of the field, and the average angular velocity Ω(*ω*)=0. In addition, the amplitude of the oscillations *θ*_*B*_(*ω*) varies inversely with the shear elastic modulus according to:


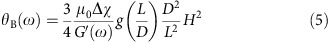


Gellan gum (phytagel, Sigma-Aldrich), a linear anionic polysaccharide comprising glucose, glucuronic acid and rhamnose building units^47^ was added slowly to a 1 mM calcium chloride solution at room temperature with rapid stirring before heating up to 50 °C. Samples prepared at concentrations 0.3, 0.5, 0.75, 1 and 2 wt. % and studied by cone-and-plate rheometry exhibited a gel-like behaviour: the elastic modulus exhibits scaling of the form *G*′(*ω*)∼*ω*^0.2^ and *G*′(*ω*) > *G*′′(*ω*) (Supplementary Note 3). At frequencies 5 × 10^−3^ to 10 rad s^−1^, the rotation angle of the wires reveal steady oscillations, and an average angular speed Ω(*ω*) equal to zero, in agreement with the Kelvin–Voigt predictions (Supplementary Fig. 8). Using equation (5), we found an elastic modulus *G*′(*ω*) of 2.5±0.8 Pa, 9.5±3 Pa and 95±30 Pa for the 0.3, 0.5 and 1 wt. % samples, respectively, again in good agreement with the cone-and-plate data (*G*′(*ω*)=3±0.5 Pa, 13±2 Pa and 130±10 Pa). In conclusion of this part, we have found that theory correctly predicts the motion of rotating magnetic wires immersed in viscoelastic model systems and that the viscoelastic parameters retrieved, such as the viscosity or the elastic modulus are those of the linear shear rheology.

### Localization of the wires inside living cells

**Figure 2:**
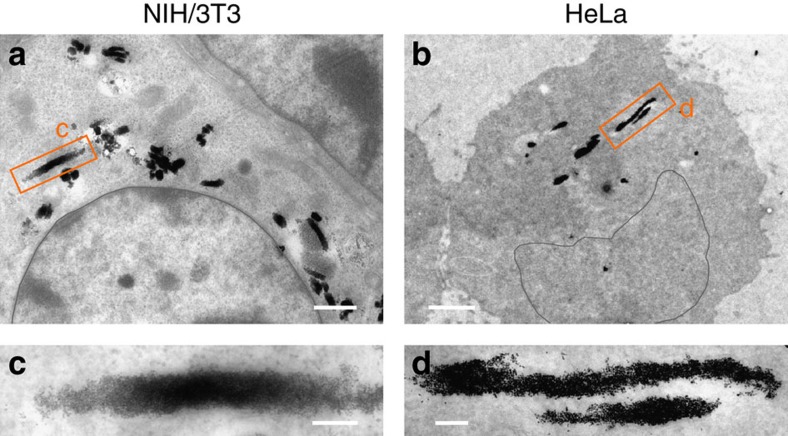
Localization of wires inside living cells. Representative transmission electron microscopy image of (**a**) NIH/3T3 fibroblasts and (**b**) HeLa cancer cells incubated with 2.4 μm wires for 24 h at the concentration of 10 wires per cell. For the fibroblasts, clusters in the range of 200 nm are also visible. Scale bars in **a**,**b** are 1 and 2 μm, respectively. (**c**,**d**) Close-up views of regions marked by an orange rectangle in **a**,**b** respectively, indicating that the wires are not enclosed in membrane-bound compartments. Scale bars are 200 nm.

Here we establish the experimental conditions under which the wires interact with the cells, and in which part of the cytoplasm they are located. The localization of the wires inside the cells was determined by transmission electron microscopy. The incubation time was 24 h and the amount of wires per cell was 10 for the two cell lines. Under such conditions, no perturbations of the cell morphology or cell cycle were observed (Supplementary Fig. 9). In a previous report, it was shown that the capture rate for neutral wires was ∼10% at 24 h, leading to an average number of one wire per cell in the present experiment^48^. Figure 2a,b display TEM images of NIH/3T3 and HeLa cell interiors, respectively. The elongated objects appearing in the TEM images are in the range 200 nm–1 μm and they are made of densely packed particles of high electronic contrast. These threads are pieces of wires that were taken up by the cells. Shorter than their initial lengths, the threads also have sharp and diffuse extremities. These observations are interpreted by assuming that elongated structures not in the plane of the microtome section were shortened during the sample preparation, an outcome that was already noted in previous studies^48^,^49^. In addition to wires, clusters in the range of 200 nm are also visible. These clusters result from the wire degradation occurring during incubation or the sample preparation. Close-up views of the TEM images in Fig. 2c,d indicate that the wires are not in membrane-bound compartments^50^. This result was already pointed out in our previous study on fibroblasts. Here however, the TEM outcomes are similar for the cancerous HeLa cells and for human lymphoblasts, indicating that the absence of endosomal membrane around internalized wires may be specific to anisotropic objects. Concerning the entry mechanism, several scenarios were suggested, including the perforation of the cellular membrane^51^,^52^, macropinocytosis^53^ or multiple combined processes^48^,^54^. At this stage, no definite conclusion can be made concerning the entry mechanism into NIH/3T3 or HeLa cells. The absence of cytoplasmic membrane around the internalized probes ensures, however, that the wires will probe the mechanical properties of the cytosol.

### Evidence of wire rotational instability in living cells

**Figure 3:**
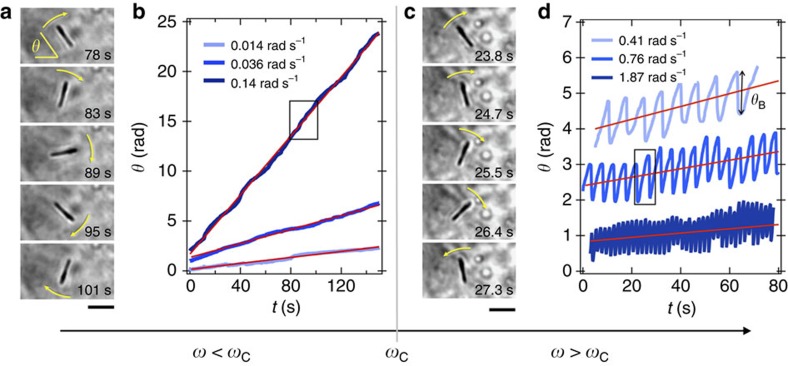
Evidence of wire rotational instability in living cells. (**a**) Optical microscopy images of a 2.8 μm internalized wire subjected to a rotating field of 14 mT and the frequency of 0.14 rad s^−1^. (**b**) Time dependences of the angle *θ*(*t*) at varying frequencies *ω*=0.014, 0.036 and 0.14 rad s^−1^. The rectangle around 80 s provides the time range for the images on the left hand side. The straight lines in red were calculated from the expression *θ*(*t*)=*ωt*, indicating that the wire rotates synchronously with the field, and at the same angular frequency. The synchronous rotation of the wire is illustrated in Supplementary Movie 1. (**c**,**d**) Same representation as in **a**,**b** but for angular frequencies (0.41, 0.76 and 1.87 rad s^−1^) above the critical frequency *ω*_C_=0.15 rad s^−1^. The microscopy images on the left-hand side shows that after a clockwise rotation, the wire comes back rapidly in an anti-clockwise motion, indicating that the wire rotation is hindered (see Supplementary Movies 2 and 3. Scale bars in **a**,**c** are 2 μm.

We then examined the motions of wires internalized into cells and submitted to a 14 mT rotating field. In this part, emphasis is put on the time-dependent behaviour. Wires of lengths 2 to 6 μm were studied as a function of the frequencies *ω*. For each condition, a 100 s movie was recorded and digitalized, from which the position of the centre-of-mass and orientation angle of the wire were retrieved and plotted as a function of time. Figure 3a shows the rotation of a 2.8 μm wire internalized into a fibroblast at the angular frequency of 0.14 rad s^−1^ and Fig. 3b illustrates the time dependence of the angle *θ*(*t*) at different frequencies (see Supplementary Movie 1). For the conditions tested, *θ*(*t*) increases linearly with time, and the slope 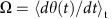 corresponds exactly to the actuating frequency. Here, 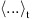 denotes the time average in the steady regime. In this first regime, the wire rotates with the field, and *θ*(*t*)=*ωt*.

Figure 3c,d illustrate the change of regime as the angular frequency is increased above a critical value, here *ω*_C_=0.15 rad s^−1^. The microscopy images on the left hand side show that after a clockwise steady rotation (between 23.8 and 26.4 s), the wire comes back rapidly by 50 degrees in an anticlockwise motion, indicating that the wire rotation is hindered. On a longer period, the wires are animated with back-and-forth motion characteristic of the asynchronous regime (Fig. 3d). The traces recorded at *ω*=0.41, 0.76 and 1.87 rad s^−1^ display steady oscillations, with a rotation angle that continues to increase with time (see Supplementary Movies 2 and 3). In this second regime, however, it is observed that 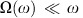. The data shown in the figure are representative of the overall behaviour found for wires in living fibroblast and HeLa cells. In these time-resolved experiments, it was verified that the centre-of-mass of the wires remained punctual during the steady or hindered rotations, insuring that the same volume of the cytosol is probed during the measurements. In conclusion to this part, with increasing frequency, wires dispersed in the intracellular medium of fibroblasts or of Hela cells undergo a transition between a synchronous and asynchronous regime. The critical frequency is found in the range 0.01–1 rad s^−1^.

### Master curve for the wire average rotation velocity

**Figure 4:**
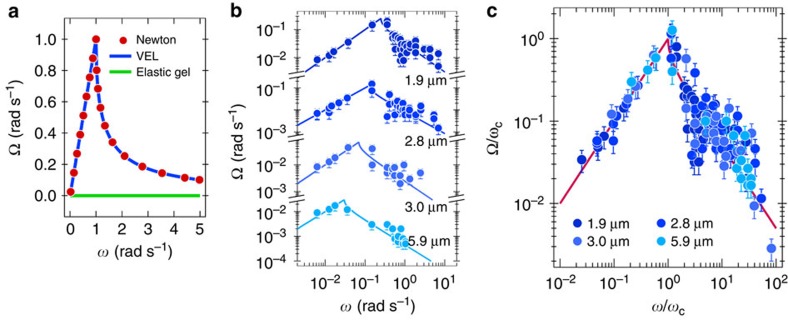
Master curve for wire average rotation velocity in cells. (**a**) Average rotation velocity Ω(*ω*) of a magnetic wire calculated for a Newtonian fluid, for a viscoelastic liquid (indicated as VEL) and for an elastic gel according to a set of models listed in Supplementary Note 1. For the sake of convenience, the viscous and the viscoelastic liquids have here the same static viscosity and *ω*_C_=1 rad s^−1^. (**b**) Ω(*ω*)-evolution for wires of lengths between 1.9 and 5.9 μm internalized in the cytoplasm of mouse fibroblasts. The continuous lines (from equation (1)) indicate the existence of a critical frequency in the rotation dynamics. (**c**) Data from Fig. 4b plotted in reduced units, Ω/*ω*_C_ versus *ω*/*ω*_C_. The continuous lines are Ω(*X*)=*X* and 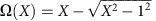 for *X*≤1 and *X≥*1, respectively. Error bars in **b**,**c** are defined as s.e.m.

To elucidate the rheological profile of NIH/3T3 and HeLa intracellular medium, the *θ*(*t*)-traces of internalized wires were analysed and translated into a set of two parameters: the average rotation velocity Ω(*ω*) and the amplitude 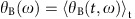 of the oscillations in the unstable regime. As mentioned in the section on the validity of the magnetic wire spectroscopy, it was found that for Newton, Maxwell and Kelvin–Voigt models, Ω(*ω*) and *θ*_B_(ω) display specific asymptotic behaviours as a function of the frequency^25^. For the viscous and viscoelastic liquids, the average frequency Ω(*ω*) is superimposed and show a cusp-like maximum at *ω*_C_, whereas for an elastic gel the average rotation velocity is constant and equal to 0. These different behaviours are illustrated in Fig. 4a.

Figure 4b displays the Ω(*ω*)-evolution for wires of lengths between 1.9 and 5.9 μm. With increasing frequency, the average velocity increases linearly, passes through a maximum at *ω*_C_ and then decreases. As already mentioned, the transition corresponds to the change of rotation regime, from synchronous rotation to back-and-forth oscillations. Note that the cut-off frequency increases as the length of the wire decreases (Fig. 4b). The data in Fig. 4 were adjusted using equation (3)^27^,^35^. In both regimes, the agreement between the data and the model is excellent. At high frequency, the Ω(*ω*)-data exhibit some scattering that could come from time-dependent effects of the viscosity, and be related to the cellular activity. Table 1 provides the values of the critical frequency and static viscosity for the experiments in Fig. 4b. There, *ω*_C_ varies from 0.03 to 0.2 rad s^−1^, and is associated to static viscosities between 20 and 80 Pa s. Figure 4c displays the data of Fig. 4b in reduced units, Ω/*ω*_*C*_ versus *ω*/*ω*_C_. In this representation, the Ω/*ω*_C_-data are found to collapse onto the same master curve, now observed over four decades in *ω*. The agreement obtained over a large frequency window attests to the reliability of the model. A comparison with the predictions shown in Fig. 4a suggests that the rheology of the intracellular medium is that of a viscous or of a viscoelastic liquid. The data in Fig. 4c also rule out the hypothesis of a gel-like rheology.

### Master curve for the wire oscillation amplitude

**Figure 5:**
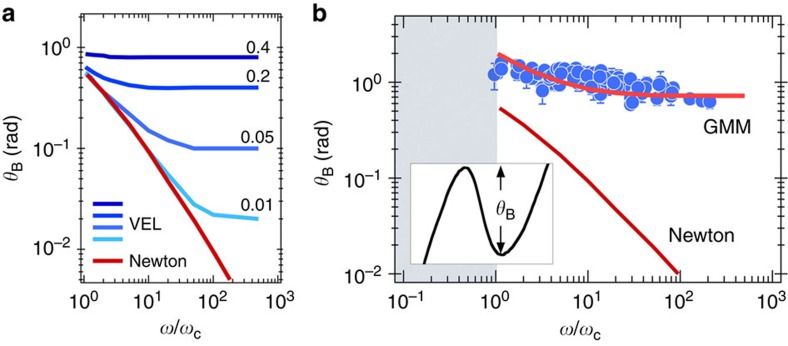
Master curve for wire oscillation amplitude in cells. (**a**) Average rotation velocity Ω(*ω*) of a magnetic wire calculated for a Newtonian fluid and for a viscoelastic liquid (indicated as VEL) of relaxation times ranging from 0.01/*ω*_C_ (light blue) to 0.4/*ω*_C_ (dark blue). (**b**) Variation of *θ*_B_ as a function of the reduced frequency *ω*/*ω*_C_ for wires of lengths 1.9 to 5.9 μm. The continuous lines were obtained from the Newtonian and from the Generalized Maxwell (GMM) models. Inset: definition of *θ*_B_. Error bars in **b** are defined as s.e.m.

Figure 5a displays the behaviours of the oscillation amplitudes for Newtonian and Maxwell fluids as a function of the reduced frequency ω/*ω*_C_. *θ*_B_ being related to the asynchronous regime, it is defined only for *ω*/*ω*_C_≥1. For the viscoelastic predictions (labelled VEL), the curves were calculated for different relaxation times ranging from 0.01/*ω*_C_ to 0.4/*ω*_C_, as indicated in the margin. For the Newtonian fluid, the amplitude of the oscillations decreases with increasing frequency. In contrast, the Maxwell fluid displays a crossover between a viscous and an elastic regime that occurs at a fixed value of the reduced frequency, here 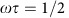. The value of 1/2 is explained by the fact that the frequency of the oscillations in this regime is twice that of the field. The existence of a plateau at high frequency is related to the elastic response of the fluid, and its height is given by equation (4) (ref. 21).

Figure 5b shows the dependence of *θ*_B_ in NIH/3T3 fibroblasts as a function of the reduced frequency for different wires of lengths between 1.9 and 5.9 μm. The major result that emerges from the figure is that the angles *θ*_*B*_ collected over many experimental conditions (that is, changing *ω* and *L*) are well superimposed when plotted against the reduced frequency. Starting at *θ*_B_=1.5 rad slightly above *ω*_C_, the angle exhibits a moderate but steady decrease with increasing *ω*. Also displayed in the figure is the prediction for a Newtonian fluid, in which decrease is much stronger and cannot account for the data found in cells. Attempts to use a single-mode Maxwell model (Fig. 5a) were also inconclusive, and the *θ*_B_-data could not be reproduced. The adjustment was noticeably improved using a Generalized Maxwell Model (noted GMM on the figure) and assuming a constant distribution of relaxation times. The spread of the distribution was found to go from 1/*ω*_C_ to 100/*ω*_C_. For *ω*_C_-values of 0.1 rad s^−1^, the time distribution extends hence from 0.1 to 10 s. From this fitting, the elastic modulus *G* of the cell interior was estimated. We found an elastic modulus *G*=12 Pa (with a s.d. of 4 Pa, *n*=9) for the fibroblasts and *G*=14 Pa (with a s.d. of 9 Pa, *n*=10) for the HeLa cells. Such values for the elasticity are in agreement with the results obtained by tracking techniques, specifically those probing the interior of the cell^13^. In contrast, *G*-values found here are one to two decades smaller than that determined by techniques probing the mechanical response of the entire cell, or that of the cell surface^4^,^5^,^9^. Such differences were already discussed in the literature, and for the latter experiments they were attributed to the cortical actin network^12^.

### Shear viscosity of the cytoplasm

**Figure 6:**
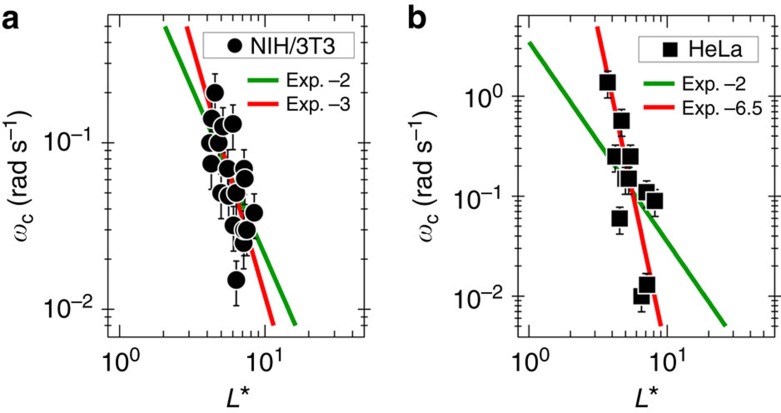
Scaling critical frequency and wire length. Variation of the critical frequency *ω*_*C*_ as a function of the reduced wire length 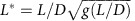 for (**a**) NIH/3T3 mouse fibroblasts and (**b**) for HeLa cervical cancer cells. Straight lines in red are least-square fits using power laws with exponent (exp.) −3 and −6.5, respectively, whereas straight lines in green are obtained from equation (2). Error bars are defined as s.e.m.

The model of rotating wires in a viscoelastic liquid contains an important prediction: the critical frequency *ω*_C_ varies inversely with the square of the wire length. This relationship was verified for fluids of known viscosity^25^,^27^ and tested again here for a 85% glycerol/water mixture (Supplementary Fig. 6). Figure 6a,b display the dependences of the critical frequency *ω*_*C*_ as a function of the reduced wire length 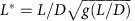 for the two cell lines studied. Least square calculations using a power law dependence of the form 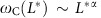 provide exponents *α*=−3.0±0.5 and *α*=−6.5±1.0 for NIH/3T3 and HeLa, respectively (straight lines in red in Fig. 6). Two related effects could account for the discrepancy with respect to equation (2): the heterogeneities of the intracellular medium, which also contribute to a relatively broad scattering of the data points, and the possible dependence of the mechanical response on the length scale. This later feature was observed in active gels of F-actin using two-particle microrheology experiments^55^. By forcing the exponent to be −2 and repeating the fitting procedure, the static shear viscosity of fibroblasts could be estimated, providing *η*_0_=47(+26/−14) Pa s. This value is larger than those reported using passive microrheology in different culture and probe conditions^13^,^21^. For the Hela cells, the deviation between the observed and predicted scaling is significant, and *η*_0_ could not be determined. This result confirms the high sampling variability of rheological parameters associated with this cell line.

## Discussion

In this work, we used magnetic wires between 2 and 6 μm to measure the static viscosity of living cells. The technique, known as rotational magnetic spectroscopy, is based on the experimental determination of a critical frequency *ω*_C_ between two different rotation regimes, one where the wire and the field are synchronous, and one where the wire performs back-and-forth oscillations. The measure of the viscosity is obtained directly from the critical frequency *ω*_*C*_, through the relationship given by equation (1). This wire-based technique is known for more than 20 years but was applied only recently to complex fluids composed of colloids of synthetic and natural origin^25^,^27^,^29^,^30^,^37^,^38^,^56^. To the best of our knowledge, it was not applied yet to living cells. In a first step, we assess the validity of the wire-based microrheology technique using known rheological models. We have found that theory and experiments agree well with each other, and that the viscoelastic parameters retrieved (including the static viscosity *η*_0_ and the elastic modulus *G*) are those of the linear shear rheology. With living cells, one of the key results here is that the rotational instability as in the range 10^−2^–1 rad s^−1^ for the wires of magnetic susceptibility *χ*=3.6. It is, therefore, accessible to standard rotating field devices mounted on an optical microscopy platform. In this paper, the analysis has been extended to the amplitude of the oscillations in the asynchronous regime and to its frequency dependence and scaling. The high frequency limit was used to estimate the shear elastic modulus *G* of the intracellular medium for the NIH/3T3 fibroblasts and for the HeLa cervical cancer cells. The modulus is in the range 5–20 Pa. The determinations of the viscosity and elasticity confirm the viscoelastic character of the cytoplasm. However here, and in stark contrast to several studies^9^,^10^,^56^, we conclude that the interior of living cells is best described in terms of a viscoelastic liquid, and not of an elastic gel. The conclusion was made possible by gaining access to a frequency range not explored before (down to 6 × 10^−3^ rad s^−1^), and for which the cytoplasm was shown to flow. The present magnetic rotational spectroscopy method does not allow to determine the distribution of relaxation times with accuracy, in part because of the restricted range of frequencies explored. An estimate is nevertheless provided and it is found that the time distribution is constant in the range 0.1–10 s (ref. 9). The rheological model used to fit the data is in agreement with a Generalized Maxwell model combined with a distribution of relaxation times. In conclusion, the present work shows the potential of the wire-based magnetic rotation spectroscopy as an accurate rheological technique to distinguish between flow and yield stress behaviours in highly confined environment.

## Methods

### Microrheology set-up

Bright field microscopy was used to monitor the actuation of the wires as a function of time. Stacks of images were acquired on an IX73 inverted microscope (Olympus) equipped with a × 100 objective. For the measure of the magnetic properties of the wires, 65 μl of a dispersion of a know viscosity were deposited on a glass plate and sealed to a Gene Frame (Abgene/Advanced Biotech) dual adhesive system. The glass plate was introduced into a homemade device (Supplementary Fig. 10) generating a rotational magnetic field, thanks to two pairs of coils working with a 90° phase shift. An electronic set-up allowed measurements in the frequency range 10^−2^–100 rad s^−1^ and at magnetic fields *B*=0–20 mT. A stream of air directed toward the measuring cell was used to thermalize the sample at *T*=26 or 37 °C. The image acquisition system consisted of an EXi Blue CCD camera (QImaging) working with Metaview (Universal Imaging Inc.). Images of wires were digitized and treated by the ImageJ software and plugins (http://rsbweb.nih.gov/ij/). The three-dimensional motion was extracted from their two-dimensional projection according a procedure described previously^26^,^44^. In brief, the out-of-plane motion of the wires is deduced from their projection on the focal plane of an optical microscope objective. An angular variable reflecting the out-of-plane motion is computed from the apparent wire length and its projected angular displacement.

### Calibration of the wire-based rheometer

To determine the susceptibility parameter Δ*χ* in equations (1) and (2), steady rotation experiments were carried out on a fluid of known viscosity. An 85 wt. % glycerol–water mixture of static viscosity *η*_0_=0.062 Pa s^−1^ (T=26 °C) was used as suspending medium. In a typical optical microscopy experiment, a wire was first selected, its length and diameter measured with the objective × 100 and it was then submitted to rotation frequency sweep from 0.1 to 100 rad s^−1^. For a statistically relevant sample, the protocol was applied on 44 wires of lengths 2–20 μm and at a magnetic field of 7 mT. In Supplementary Fig. 10, *ω*_C_ is shown as a function of L*. The critical frequency was found to decrease as 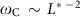, in agreement with the prediction of equation (2). From the prefactor (3*μ*_0_Δ*χH*^2^/8*η*_0_=590±180 rad s^−1^), we infer Δ*χ*=2.3±0.7, and *χ*=3.6±0.9. Knowing Δ*χ*, a wire submitted to a rotating field will be subjected to a known magnetic torque, a condition that is a prerequisite for quantitative rotational microrheology.

### Cell culture

NIH/3T3 fibroblast (ATCC-CRL-1658) and HeLa (ATCC-CCL-2) cells were grown in T25-flasks as a monolayer in DMEM with high glucose (4.5 g l^−1^) and stable glutamine (PAA Laboratories GmbH, Austria). The medium was supplemented with 10% fetal bovine serum and 1% penicillin/streptomycin (PAA Laboratories GmbH), referred to as cell culture medium. Exponentially growing cultures were maintained in a humidified atmosphere of 5% CO_2_ and 95% air at 37 °C, and in these conditions, the plating efficiency was 70–90% and the cell duplication time was 12–14 h. Cell cultures were passaged twice weekly using trypsin–EDTA (PAA Laboratories GmbH) to detach the cells from their culture flasks and wells. The cells were pelleted by centrifugation at 260 *g* for 5 min. The supernatant was removed and cell pellets were re-suspended in assay medium and counted using a Malassez counting chamber.

### Transmission electron microscopy

NIH/3T3 fibroblast cells were seeded onto the six-well plate. After a 24-h incubation with 4 μm wires, the excess medium was removed. The cells were washed in 0.2 M phosphate buffer (phosphate-buffered saline, PBS) and fixed in 2% glutaraldehyde-phosphate buffer (0.1 M) for 1 h at room temperature. Fixed cells were further washed in 0.2 M PBS. The cells were then postfixed in a 1% osmium-phosphate buffer for 45 min at room temperature in dark conditions. After several washes with 0.2 M PBS, the samples were dehydrated by addition of ethanol. Samples were infiltrated in 1:1 ethanol:epon resin for 1 h and finally in 100% epon resin for 48 h at 60 °C for polymerization. Ninety-nanometre-thick sections were cut with an ultramicrotome (LEICA, Ultracut UCT) and picked up on copper–rhodium grids. They were then stained for 7 min in 2%-uranyl acetate and for 7 min in 0.2%-lead citrate. Grids were analysed with a transmission electron microscope (ZEISS, EM 912 OMEGA) equipped with a LaB_6_ filament, at 80 kV. Images were recorded with a digital camera (SS-CCD, Proscan 1024 × 1024), and the iTEM software.

## Additional information

**How to cite this article:** Berret, J.-F. *et al.* Local viscoelasticity of living cells measured by rotational magnetic spectroscopy. *Nat. Commun.* 7:10134 doi: 10.1038/ncomms10134 (2016).

## Supplementary Material

Supplementary Information Supplementary Figures 1-10, Supplementary Table 1, Supplementary Notes 1-3 and Supplementary References

Supplementary Movie 1 Representing the synchronous rotation of a 2.8 μm long wire internalized in a NIH/3T3 fibroblast cell at the angular frequency of 0.146 rad s^-1^, under an applied magnetic field of 14 mT. The wire rotates at the same angular frequency as the field.

Supplementary Movie 2 Representing the asynchronous rotation of a 2.8 μm long wire internalized in a NIH/3T3 fibroblast cell at the angular frequency of 1.18 rad s^-1^, under an applied magnetic field of 14 mT. The wire exhibits a back and forth motion, indicating that the frequency is above the critical frequency (here 0.15 rad s^-1^)

Supplementary Movie 3 Representing the asynchronous rotation of a 2.8 μm long wire internalized in a NIH/3T3 fibroblast cell at the angular frequency of 3.78 rad s^-1^, under an applied magnetic field of 14 mT. The wire exhibits a back and forth motion, indicating that the frequency is above the critical frequency (here 0.15 rad s^-1^)

## Figures and Tables

**Table 1:** Viscoelastic parameters of NIH/3T3 fibroblast.

**Wire length**	***ω***_**C**_ **(rad** **s**^−1^**)**	***η***_**0**_ **(Pa** **s)**	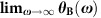 **(rad)**	***G*** **(Pa)**
*L*=1.9 μm	0.20±0.05	32±8	0.8±0.1	16±2
*L*=2.8 μm	0.15±0.02	26±5	0.8±0.1	9±2
*L*=3.0 μm	0.07±0.02	59±15	0.8±0.1	11±2
*L*=5.9 μm	0.03±0.01	78±20	0.6±0.1	8±2

*ω*_C_ denotes the critical cut-off frequency between the synchronous and asynchronous regimes for the data in Fig. 4b. *η*_0_ is the static viscosity derived from equation (1), 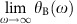 is the angle by which the wire returns back at high frequency and *G* is the elastic modulus obtained from equation (4). The error bars are defined as s.e.m.
