# Supplementary material for: Local viscoelasticity of living cells measured by rotational magnetic spectroscopy
Source: Nat Commun. 2016 Jan 5;7:10134. doi: 10.1038/ncomms10134 (PMC4728338; doi:10.1038/ncomms10134)
Supplement: Supplementary Information — Supplementary Figures 1-10, Supplementary Table 1, Supplementary Notes 1-3 and Supplementary References [file ncomms10134-s1.pdf]

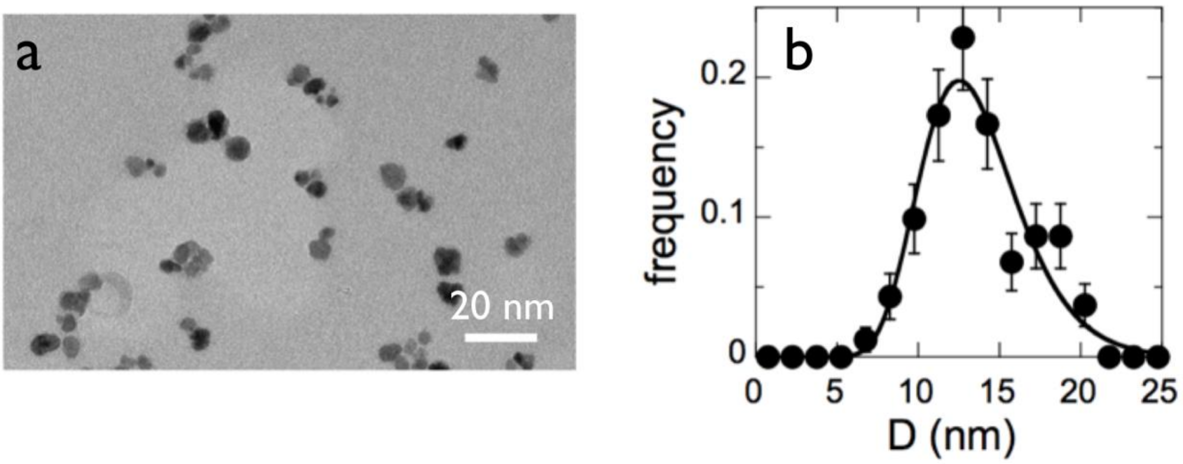

Supplementary Figure 1 - Transmission electron microscopy (TEM)
a) TEM image of iron oxide nanoparticles. b) Size distribution.

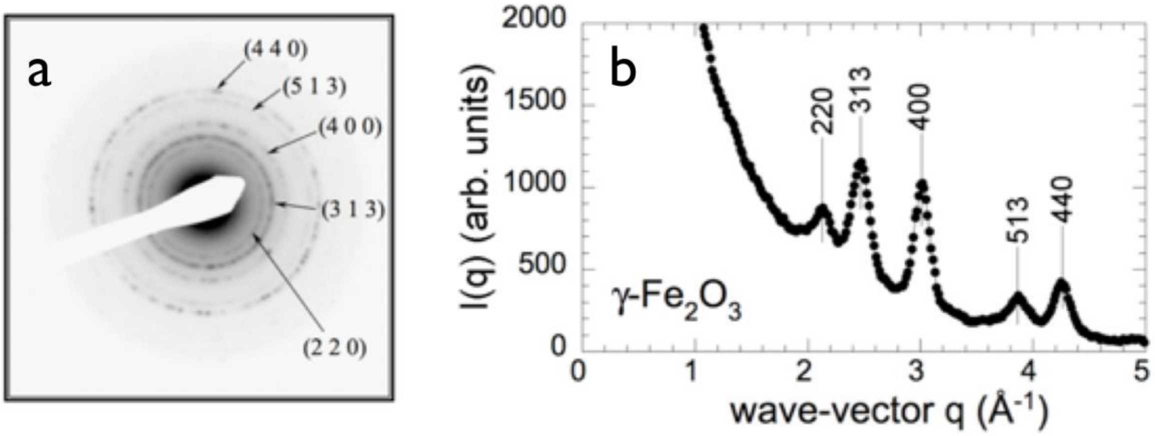

Supplementary Figure 2 - Electron beam microdiffraction
a) Microdiffraction spectrum of iron oxide nanoparticles. b) Wave-vector dependence of the scattering intensity obtained from a). The Bragg peaks observed are associated to the structure of maghemite.

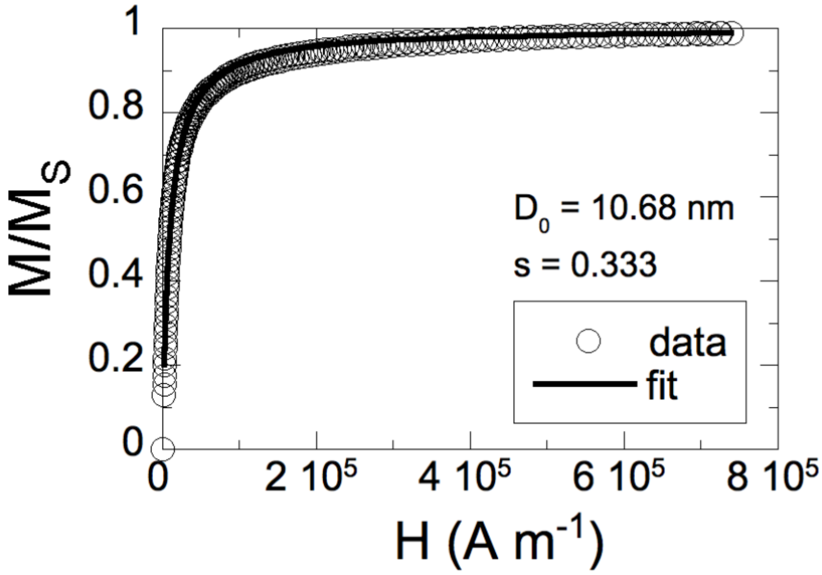

Supplementary Figure 3 - Vibrating sample magnetometry:
Magnetic field dependence of the macroscopic magnetization $M(H)$ normalized by its saturation value M_S for cationic maghemite dispersions. The solid curve was obtained using the Langevin equation convoluted with a log-normal distribution of particle sizes. Here, $M_S = \phi m_S$ where m_S is the specific magnetization of colloidal maghemite ($m_S = 3.5 \times 105\ \mathrm{A}\ m^{-1}$) and ϕ the volume fraction.

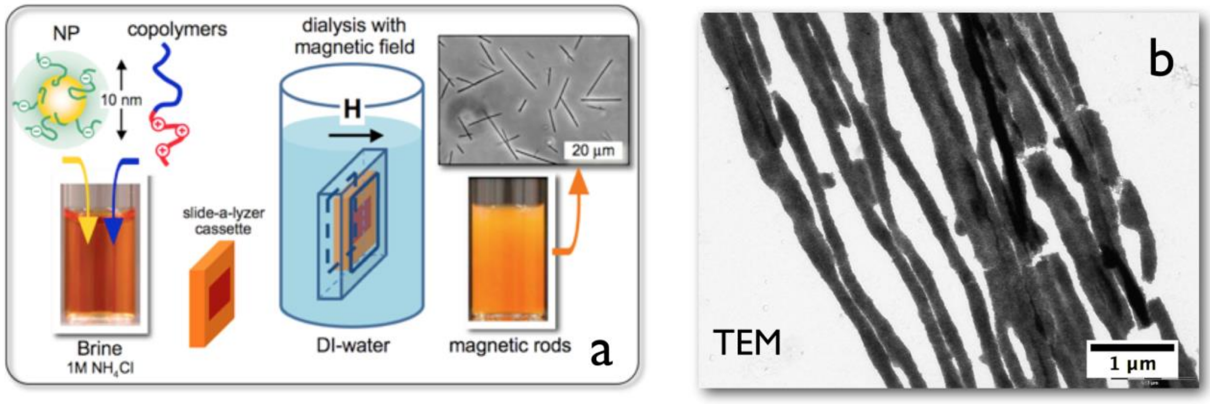

Supplementary Figure 4 - **Fabrication of nanostructured wires**
a) Schematic representation of the protocol that controls the nanoparticle co-assembly and wire formation. The dialysis involves the preparation of separate 1 M NH_4Cl salted solutions of particles and copolymers. The ionic strength is progressively diminished by dialysis with a 10000 g mol^{-1} cut-off Slide-a-Lyzer® cassette. b) Image of wires aggregated on a TEM grid.

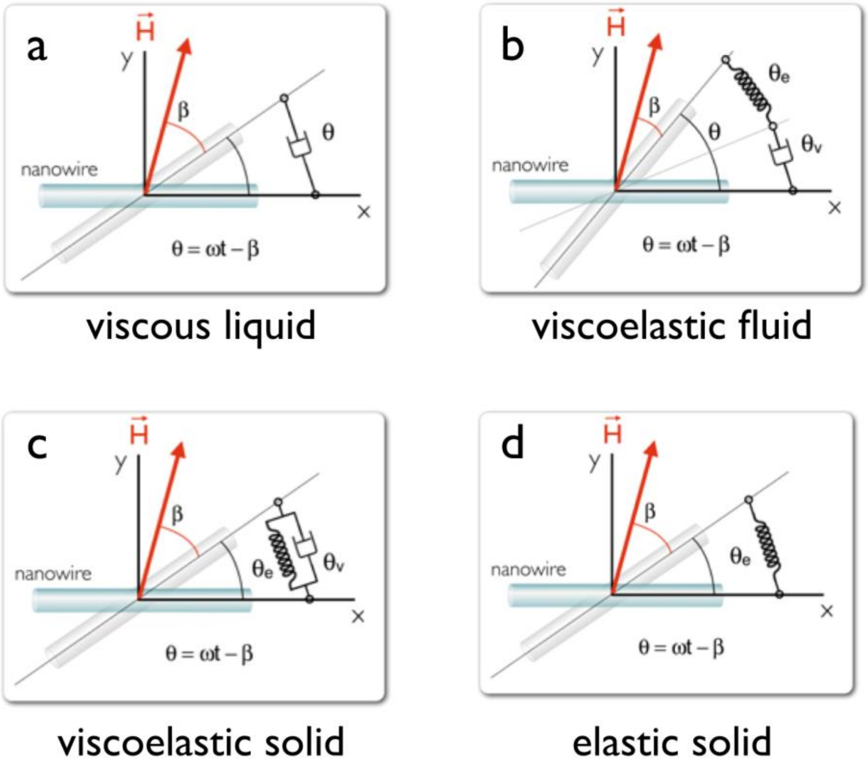

Supplementary Figure 5 – Wire rotation in model rheological fluids and solids
Schematic representation of a wire in a purely viscous liquid (a), in a viscoelastic liquid (b), in a viscoelastic solid (c) and in an elastic solid (d). The viscous liquid is shown as a dashpot and the elastic solid as a spring. A spring and a dashpot in series form a Maxwell element. A spring and a dashpot in parallel form a Kelvin-Voigt element.

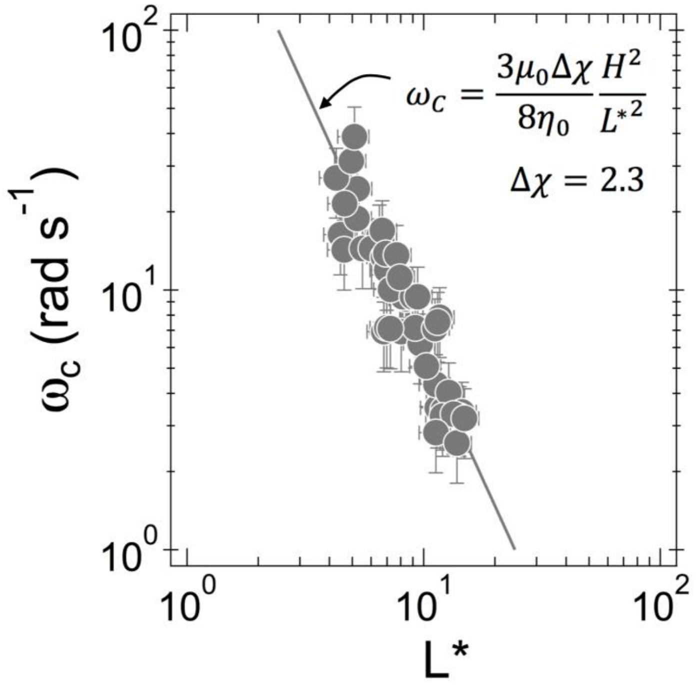

Supplementary Figure 6 - Determination of the wire magnetic susceptibility

Critical frequency ω_C as a function of the parameter $L^* = L/D\sqrt{g(L/D)}$ obtained for wires dispersed in a 85 wt. % glycerol-water mixture of know viscosity. The straight line is calculated using Eq. 2 and $\Delta\chi = \chi^2/(2+\chi) = 2.3 \pm 0.4$.

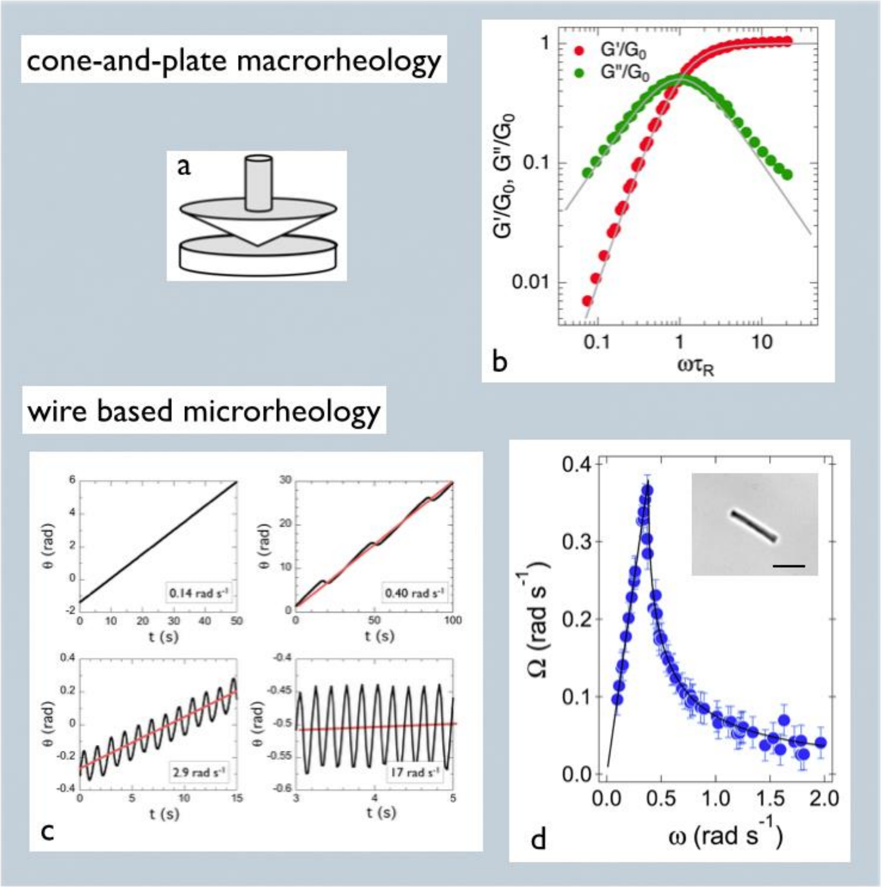

Supplementary Figure 7 - **Microrheology on a model viscoelastic liquid**
a) Cone-and-plate geometry used in shear rheometry. b) $G'(\omega)/G$ and $G''(\omega)/G$ versus frequency for a wormlike micellar fluid. The continuous lines are Maxwell predictions. c) Rotation angle $\theta(t)$ of a 8.1 µm wire as a function of the time at 0.14, 0.40, 2.9 and 17.0 rad s^{-1}. d) Average angular velocity $\Omega(\omega)$ as a function of the frequency. The solid line corresponds to the best fit using Eq. 3 (main text). Inset in d): image of the wire by phase contrast microscopy (60×, scale bar 5 µm).

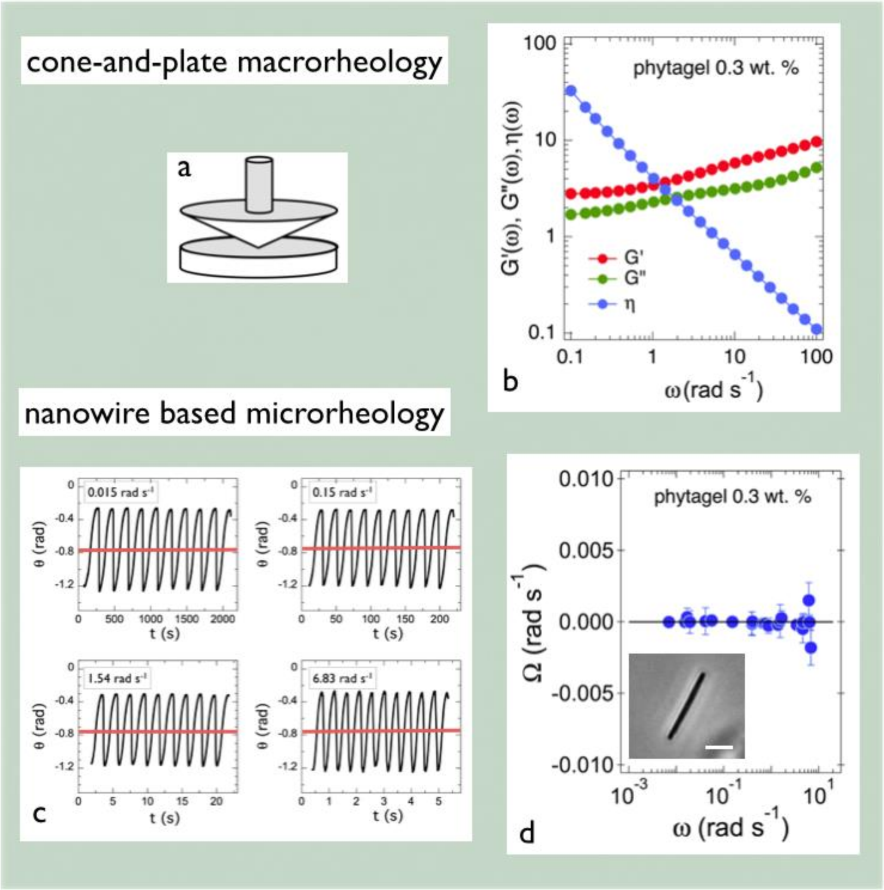

Supplementary Figure 8 - **Microrheology on a model viscoelastic gel**
a) Cone-and-plate geometry used in shear rheometry. b) $G'(\omega)$, $G''(\omega)$ and $\eta(\omega)$ versus frequency of a 0.3 wt. % gellan gum (phytagel) sample. c) Rotation angle $\theta(t)$ of a 12 µm wire as a function of the time at 0.015, 0.15, 1.54 and 6.83 rad s^{-1}. d) Average angular velocity $\Omega(\omega)$ as a function of the frequency. The solid line corresponds to the best fit using the Kelvin-Voigt model. Inset in d): image of the 12 µm wire by phase contrast microscopy (60×, scale bar 5 µm).

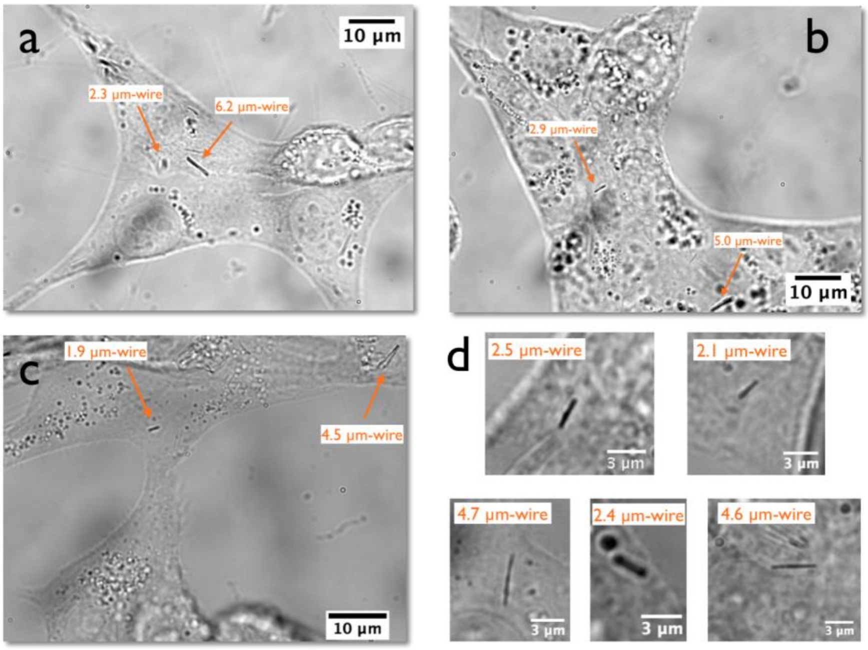

Supplementary Figure 9 – wires inside cells
a - d) Examples of wires internalized into NIH/3T3 fibroblasts. These wires were studied by magnetic rotational spectroscopy. Their lengths vary from 1.9 µm to 6.2 µm.

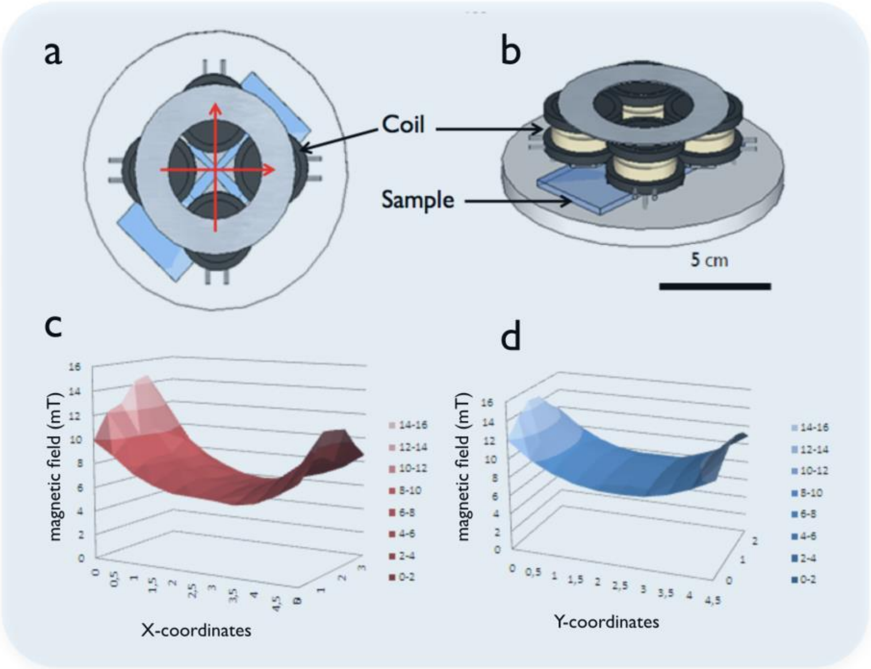

Supplementary Figure 10 - Magnetic field rotating device
Top (a) and side (b) views of the rotating field device used this work. Magnetic field distributions are shown along the X (c) and Y (d) axis of the four-coil device. In the center, the magnetic field is constant over a 1×1 mm^2 range.

nanoparticles	zeta potential ζ (mV)	electrophoretic mobility μ_E (10^{-4} cm^2 V^{-1})
Fe_2O_3 (bare)	+ 40	+ 3.1
PAA_{2K}- γ -Fe_2O_3	- 48	- 3.8

Supplementary Table 1 - **Electrophoretic mobility and zeta potential**
Zeta potential (ζ) and electrophoretic mobility (μ_E) for bare and coated iron oxide particles measured using laser Doppler velocimetry and phase analysis light scattering method (Zetasizer NanoZS, Malvern Instrument).

Supplementary Note 1 - Equations of motions of wires in various model fluids and solids

Newton Model (viscous liquid)

The basic hypotheses are given in the paper by Frka-Petesic *et al.*[2]. The configuration of a wire subjected to a rotational field is shown in Supplementary Figure 5a. Under steady rotation, the expression of the magnetic torque reads :

$$\Gamma_m(H) = \frac{\chi^2}{2(2+\chi)}\mu_0 V H_0^2 \sin(2\beta)$$

where $V = \pi D^2 L/4$ is the volume of the wire, χ the magnetic susceptibility. The viscous torque reads:

$$\Gamma_v = -\frac{\pi L^3}{3g(\frac{L}{D})}\,\eta_0 \frac{d\theta}{dt}$$

The balance between the two torques gives the differential equation:

$$\frac{d\theta}{dt} = \omega_C \sin 2(\omega t - \theta)$$

where

$$\omega_C = \frac{3}{8}\frac{\mu_0 \Delta\chi}{\eta} g(\frac{L}{D})\frac{D^2}{L^2} H_0^2$$

and $\theta = \omega t - \beta$

The above differential equation can be expressed in therms of the variable β, leading to:

$$\frac{d\beta}{dt} = \omega - \omega_C \sin 2\beta$$

In the stationary state, $\frac{d\beta}{dt} = 0$ and two regimes can be obtained:

Regime 1: $\boldsymbol{\omega < \omega_C}$: In this case, it is possible to find a steady solution for the wire orientation, as $\sin 2\beta < 1$. β is then defined as $\frac{1}{2} Arcsin(\frac{\omega}{\omega_C})$. The wire rotation is here synchronous with the field, and the wire exhibits a delay with respect to the field.

Regime 2: $\boldsymbol{\omega > \omega_C}$: In this case, it is not possible to find a staionary value of β as $\sin 2\beta > 1$. In this regime the rotation is asynchronous. The critical frequency ω_C separates two regimes, one of steady rotation at low frequency, and one of back-and-forth motions at high frequency.

Maxwell Model (viscoelastic liquid)

The magnetic wire is subjected to a rotational field and the fluid is represented by a Maxwell element (Supplementary Figure 5b)[3]. The expression of the magnetic torque is again given by:

$$\Gamma_m(H) = \frac{\chi^2}{2(2+\chi)}\mu_0 V H_0^2 \sin(2\beta)$$

where $V = \pi D^2 L/4$ is the volume of the wire, χ the magnetic susceptibility. Immersed in a viscoelastic Maxwell fluid, a wire experiences two restoring torques that slow down its rotation. One torque has a viscous origin and writes:

$$\Gamma_v = \frac{\pi\,\eta_0 L^3}{3g\left(\frac{L}{D}\right)}\frac{d\theta_v}{dt}$$

and one torque has an elastic origin and writes:

$$\Gamma_e = \frac{\pi G L^3}{3g\left(\frac{L}{D}\right)}\theta_e$$

In the previous equations, η_0 is the static viscosity of the fluid, G its elastic modulus and $g\left(\frac{L}{D}\right)$ is a dimensionless function of the anisotropy ratio $p = L/D$. In this study, we assume that[4]:

$$g(p) = \ln(p) - 0.662 + 0.917/p \ - 0.050/p^2$$

The previous equation is valid in the interval $2 < p < 20$. For a Maxwell fluid, $\eta_0 = G\tau$ with τ is the relaxation time. In rheology, the Maxwell model is depicted as a spring and a dashpot in series. In this configuration, the elastic and viscous deformations are additive, and the shear stresses are equal. By analogy, we assume here that $\theta = \theta_v + \theta_e$, and that $\Gamma_m = \Gamma_v = \Gamma_e$, resulting in the differential equation:

$$\frac{d\theta(t)}{dt}(1 + \theta_0 \cos 2(\omega t - \theta)) = \omega_C sin2(\omega t - \theta) + \omega\theta_0 \cos 2(\omega t - \theta)$$

where

$$\omega_C = \frac{3}{8}\frac{\mu_0 \Delta\chi}{\eta_0} g\left(\frac{L}{D}\right)\frac{D^2}{L^2}H^2$$

and

$$\theta_0 = \frac{3}{4}\frac{\mu_0 \Delta\chi}{G_0} g(\frac{L}{D})\frac{D^2}{L^2}H^2$$

In the previous equations, the two quantities ω_C and θ_0 vary quadratically with the magnetic excitation and $\theta_0 = 2\omega_C\tau$. Here, τ denotes the characteristic relaxation time of the fluid ($\tau = \eta_0/G$). The differential equation was solved using the MAPPLE software, which allows to derive the rotation phase behavior of a wire in a Maxwell fluid[3].

Kelvin-Voigt Model (viscoelastic solid)

The definitions are identical to those of the previous section and the solid is here represented by a Kelvin-Voigt element (Supplementary Figure 5c). The expression of the magnetic torque is :

$$\Gamma_m(H) = \frac{\chi^2}{2(2+\chi)}\mu_0 V H_0^2 \sin(2\beta)$$

where V is the volume of the wire, χ the magnetic susceptibility. Immersed in a Kelvin-Voigt fluid, a wire experiences two restoring torques that slow down its rotation. One torque has a viscous origin and writes:

$$\Gamma_v = \frac{\pi\,\eta_0 L^3}{3g\left(\frac{L}{D}\right)}\frac{d\theta_v}{dt}$$

and one torque has an elastic origin and writes:

$$\Gamma_e = \frac{\pi G L^3}{3g\left(\frac{L}{D}\right)}\theta_e$$

η_0 is the static viscosity associated to the dashpot and G is the modulus of the elastic spring. $g\left(\frac{L}{D}\right)$ is a dimensionless function defined previously. In rheology, the Kelvin-Voigt model is depicted as a spring and a dashpot in parallel. In this configuration, the torques are additive, and the deformations are equal. By analogy, we assume here that $\theta = \theta_v = \theta_e$, and that $\Gamma_m = \Gamma_v + \Gamma_e$, resulting in the differential equation:

$$\tau \frac{d\theta(t)}{dt} + \theta = \frac{\theta_0}{2}\sin 2(\omega t - \theta)$$

where

$$\theta_0 = \frac{3}{4}\frac{\mu_0 \Delta\chi}{G} g(\frac{L}{D})\frac{D^2}{L^2}H^2$$

In the previous expression, the viscosity does not show up, which is expected for a system for which this quantity is not defined. The differential equation was solved using the MAPPLE software.

Hooke Model (elastic solid)

The definitions are identical to those of the previous section and the solid is represented by a spring (Supplementary Figure 5d). The expression of the magnetic torque is :

$$\Gamma_m(H) = \frac{\chi^2}{2(2+\chi)}\mu_0 V H_0^2 \sin(2\beta)$$

where V is the volume of the wire, χ the magnetic susceptibility. Immersed in a Hookean solid, a wire experiences a torque of the form:

$$\Gamma_e = \frac{\pi G L^3}{3g\left(\frac{L}{D}\right)}\theta_e$$

G is the modulus of the elastic spring. $g\left(\frac{L}{D}\right)$ has the same definition as above. The balance between the two torques gives:

$$\theta = \frac{\theta_0}{2}\sin 2(\omega t - \theta)$$

where

$$\theta_0 = \frac{3}{4}\frac{\mu_0 \Delta\chi}{G} g(\frac{L}{D})\frac{D^2}{L^2}H^2$$

For the Hookean solid, the modulus is the equilibrium modulus, i.e. the modulus at long time.

Supplementary Note 2 - Microrheology on a viscoelastic model fluid: the case of a wormlike micellar solution

To assess the validity of the wire-based microrheology approach, magnetic wires were tested on several model fluids and gels: water-glycerol mixtures, wormlike micellar solutions and polysaccharide gels. Here we discuss the case of the surfactant solution, and compare the results of macro- and microrheology[3].

The solution studied is a mixture of cetylpyridinium chloride and sodium salicylate, abbreviated as CPCl/NaSal dispersed in a 0.5 M NaCl brine at the concentration of 2 wt.%. CPCl and NaSal are known to self-assemble spontaneously into micrometer long wormlike micelles, which then build a semi-dilute entangled network (as regular polymers) above a certain concentration[5-7]. This network confers to the solution a viscoelastic behavior. For CPCl/NaSal solutions, the viscoelasticity is of very peculiar type, as these fluids appear to be perfect Maxwell fluids. At the concentration of 2 wt.%,

the mesh size of the network is of the order of 30 nm, *i.e.* much smaller than the wire diameter. Surfactant wormlike micelles are sometimes called equilibrium polymers.

Cone-and-plate macrorheology Supplementary Figure 7a displays a cone-and-plate device (diameter 50 mm, angle of the cone 2°) used in macrorheology and Supplementary Figure 7b the frequency dependence of the elastic and loss moduli $G'(\omega)$ and $G''(\omega)$ obtained on the 2 wt.% solution. Data were obtained on a CSL 100 rheometer (TA Instruments). At $T = 27\ °C$, this wormlike micellar fluid was characterized by the static viscosity $\eta_0 = 1.0 \pm 0.1$ Pa s, a relaxation time $\tau = 0.14 \pm 0.01$ s and an elastic modulus $G = 7.1 \pm 0.1$ Pa, in agreement with earlier literature[5-7]. In Supplementary Figure 7b, the $G'(\omega)$ and $G''(\omega)$ data were normalized with respect to the elastic modulus and the frequency with respect to the relaxation time. The continuous lines are the predictions for a Maxwell fluid: $G'(X)/G = X^2/(1+X^2)$ and $G''(\omega)/G = X/(1+X^2)$, where $X = \omega\tau$. The agreement between the data and predictions is excellent.

Wire-based microrheology In Supplementary Figures 7c and 7d, a rotating magnetic field of 10.4 mT was applied to a 8.1 µm nanowire (inset) immersed in the CPCl/NaSal solution at increasing frequencies, between 0.1 and 20 rad s^{-1}. The motion of the wires was monitored by optical microscopy, and the time dependence of their orientation was derived. Supplementary Figure 7c show 4 experimental time traces $\theta(t)$ obtained at $\omega =$ 0.14, 0.40, 2.9 and 17.0 rad s^{-1}. At low frequency, the wire rotates with the field, and $\theta(t) = \omega t$. Above the critical frequency (here $\omega_C = 0.38\ \mathrm{rad\ s^{-1}}$), the wire is animated of back-and-forth motion characteristic of the asynchronous regime. In this range, $\theta(t)$ displays oscillations. The red straight lines in the figures represent the average angular velocity $\Omega(\omega)$.

Supplementary Figure 7d displays the evolution of the average angular velocity $\Omega(\omega)$ versus ω obtained for $L = 8.1\ \mu m$. With increasing frequency, $\Omega(\omega)$ increases, passes through a cusp-like maximum at the critical frequency ($\omega = \omega_C$), and then decreases. The $\Omega(\omega)$-data were adjusted using Eq. 1 (main text) and a value of the static viscosity of $\eta_0 = 1.3 \pm 0.3$ Pa s, in good agreement with the cone-and-plate rotational rheometry value at this temperature, $\eta_0 = 1.0 \pm 0.1$ Pa s. Experiments performed with wires in different conditions confirmed the above values. The comparison between macro- and microrheology shows that rotating wires are able to account for the static shear viscosity and the shear elasticity of a Maxwell fluid.

Supplementary Note 3 - Microrheology on a gel: the case of a polysaccharide phytagel

Gellan gum, also known as phytagel (Sigma-Aldrich) is a linear anionic heteropolysaccharide of molecular weight 5×10^5 Da, which comprises a monosaccharide building units glucose, glucuronic acid and rhamnose in a molar ratio of 2:1:1. Gellan gum is an extracellular polysaccharide produced by the bacteria *P. elodea*. Divalent cations such as calcium or magnesium are required to stabilize and cross-link the gellan three-dimensional network. Thanks to its remarkable room

temperature gelifying properties, phytagel enters into many fabricated food applications including confectioneries, jellies, fillings and dairy products. Phytagel is also intended for use in place of agar and other gelling agents for plant tissue culture[8].

Phytagel powder was added slowly to a 1 mM calcium chloride solution at room temperature with rapid stirring to eliminate any lumps before heating up to 50 °C. Samples prepared at concentrations 0.1, 0.2, 0.3, 0.5, 0.75, 1 and 2 wt. % were studied by cone-and-plate rheometry in the same conditions as the surfactant micelles. The sol-gel transition was found between 0.2 and 0.3 wt. %.

Cone-and-plate macrorheology Supplementary Figure 8a displays the cone-and-plate geometry used for the rheological measurements and Supplementary Figure 8b the frequency dependences of the elastic and loss moduli $G'(\omega)$ and $G''(\omega)$ for the 0.3 wt. % sample. There, $G'(\omega)$ and $G''(\omega)$ exhibit scaling behaviors with exponents 0.20 and 0.15 respectively. In addition, on the whole frequency range, the inequality $G'(\omega) > G''(\omega)$ is found. These two properties are known as being strong evidences of the gel-like character of the sample[8,9]. Similar rheological results were found at 0.5, 0.75 and 1 wt. %.

Wire-based microrheology For microrheology, wires of length 6 to 45 µm were immersed in phytagel samples at various concentrations (0.3, 0.5 and 1 wt. %) and submitted to a rotating magnetic field of 12 mT at a frequency between 5×10^{-3} and 10 rad s^{-1}. Supplementary Figure 8c shows time traces of the rotation angle obtained at $\omega =$ 0.015, 0.15, 1.54 and 6.83 rad s^{-1} in the 0.3 wt. % phytagel solution. Over 3 decades in frequency, the traces reveal a unique behavior: $\theta(t)$ displays regular oscillations at a frequency double of that of the field, and an average angular velocity Ω (shown as red straight lines) close to zero. Similar results were found at 0.5 and 1 wt. %. Supplementary Figure 8d displays the evolution of the average angular velocity $\Omega(\omega)$ versus ω, and indicates that for all frequency tested $\Omega(\omega) = 0$ within the measurement uncertainty. This behavior is in agreement with the prediction for the Kelvin-Voigt described in Suplementary Note 1.

From the amplitude of the oscillation $\theta_B(\omega)$, the elastic modulus $G'(\omega)$ was estimated using the expression:

$$G'(\omega) = \frac{3}{4}\frac{\mu_0 \Delta\chi}{\theta_B(\omega)} g(\frac{L}{D}) \frac{D^2}{L^2} H^2$$

In the low frequency range, we found $G' = 2.5 \pm 0.8$ Pa, 9.5 ± 3 Pa and 95 ± 30 Pa for the 0.3, 0.5 and 1 wt. % samples, respectively. These values are in good agreement with the rheological cone-and-plate data ($G' = 3 \pm 0.5$ Pa, 13 ± 2 Pa and 130 ± 10 Pa). In conclusion, we show that the wire-based microrheology is able to account for the time and frequency dependencies of a gel of Kelvin-Voigt type.

Supplementary References

1. Berret J-F, Sandre O, Mauger A. Size distribution of superparamagnetic particles determined by magnetic sedimentation. *Langmuir* **23**, 2993-2999 (2007).

2. Frka-Petesic B*, et al.* Dynamics of paramagnetic nanostructured rods under rotating field. *J Magn Magn Mater* **323**, 1309-1313 (2011).

3. Chevry L, Sampathkumar NK, Cebers A, Berret JF. Magnetic wire-based sensors for the microrheology of complex fluids. *Phys Rev E* **88**, (2013).

4. Tirado MM, Martinez CL, Delatorre JG. Comparison of theories for the translational and rotational diffusion-coefficients of rod-like macromolecules - application to short DNA fragments. *J Chem Phys* **81**, 2047-2052 (1984).

5. Berret J-F, Appell J, Porte G. Linear rheology of entangled wormlike micelles. *Langmuir* **9**, 2851-2854 (1993).

6. Berret J-F. Transient rheology of wormlike micelles. *Langmuir* **13**, 2227-2234 (1997).

7. Berret J-F, Porte G, Decruppe JP. Inhomogeneous shear rows of wormlike micelles: A master dynamic phase diagram. *Phys Rev E* **55**, 1668-1676 (1997).

8. Nussinovitch A. *Hydrocolloid Applications: Gum technology in the food and other industries* Springer Science+Business Media Dordrecht (1997).

9. Larson RG. *The Structure and Rheology of Complex Fluids*. Oxford University Press (1998).
